# Molecular Mechanisms of Anticancer Activity of N-Glycosides of Indolocarbazoles LCS-1208 and LCS-1269

**DOI:** 10.3390/molecules26237329

**Published:** 2021-12-02

**Authors:** Roman G. Zenkov, Olga A. Vlasova, Varvara P. Maksimova, Timur I. Fetisov, Natalia Y. Karpechenko, Lidiya V. Ektova, Vera A. Eremina, Valeriia G. Popova, Olga G. Usalka, Ekaterina A. Lesovaya, Gennady A. Belitsky, Marianna G. Yakubovskaya, Kirill I. Kirsanov

**Affiliations:** 1 N. N. Blokhin Russian Cancer Research Center, 24 Kashirskoe Shosse, 115478 Moscow, Russia; olya_vlasov@mail.ru (O.A.V.); lavarvar@gmail.com (V.P.M.); TimkaTryam@yandex.ru (T.I.F.); nojadg@mail.ru (N.Y.K.); lidia.ektova@yandex.ru (L.V.E.); vera.eremina2019@gmail.com (V.A.E.); nuarrbio@gmail.com (V.G.P.); ousalka@mail.ru (O.G.U.); lesovenok@yandex.ru (E.A.L.); belitsga@mail.ru (G.A.B.); mgyakubovskaya@mail.ru (M.G.Y.); kkirsanov85@yandex.ru (K.I.K.); 2 Faculty of Biotechnology and Industrial Ecology, Mendeleev University of Chemical Technology of Russia, 9 Miusskaya Ploshchad, 125047 Moscow, Russia; 3 International School “Medicine of the Future”, I.M. Sechenov First Moscow State Medical University, 8-2 Trubetskaya St., 119991 Moscow, Russia; 4 Department of Oncology, I.P. Pavlov Ryazan State Medical University, 9 Vysokovoltnaya St., 390026 Ryazan, Russia; 5 Institute of Medicine, RUDN University, 6 Miklukho-Maklaya St., 117198 Moscow, Russia

**Keywords:** LCS-1208, LCS-1269, indolocarbazoles, antitumor activity, intercalation, chromatin remodeling, interferon, epigenetics

## Abstract

Novel indolocarbazole derivatives named LCS were synthesized by our research group. Two of them were selected as the most active anticancer agents in vivo. We studied the mechanisms of anticancer activity in accordance with the previously described effects of indolocarbazoles. Cytotoxicity was estimated by MTT assay. We analyzed LCS-DNA interactions by circular dichroism in cholesteric liquid crystals and fluorescent indicator displacement assay. The effect on the activity of topoisomerases I and II was studied by DNA relaxation assay. Expression of interferon signaling target genes was estimated by RT-PCR. Chromatin remodeling was analyzed–the effect on histone H1 localization and reactivation of epigenetically silenced genes. LCS-induced change in the expression of a wide gene set was counted by means of PCR array. Our study revealed the cytotoxic activity of the compounds against 11 cancer cell lines and it was higher than in immortalized cells. Both compounds bind DNA; binding constants were estimated—LCS-1208 demonstrated higher affinity than LCS-1269; it was shown that LCS-1208 intercalates into DNA that is typical for rebeccamycin derivatives. LCS-1208 also inhibits topoisomerases I and IIα. Being a strong intercalator and topoisomerase inhibitor, LCS-1208 upregulates the expression of interferon-induced genes. In view of LCSs binding to DNA we analyzed their influence on chromatin stability and revealed that LCS-1269 displaces histone H1. Our analysis of chromatin remodeling also included a wide set of epigenetic experiments in which LCS-1269 demonstrated complex epigenetic activity. Finally, we revealed that the antitumor effect of the compounds is based not only on binding to DNA and chromatin remodeling but also on alternative mechanisms. Both compounds induce expression changes in genes involved in neoplastic transformation and target genes of the signaling pathways in cancer cells. Despite of being structurally similar, each compound has unique biological activities. The effects of LCS-1208 are associated with intercalation. The mechanisms of LCS-1269 include influence on higher levels such as chromatin remodeling and epigenetic effects.

## 1. Introduction

One of the key directions in modern anticancer therapy is the search for antitumor agents among those small molecules that noncovalently bind to nucleic acids. These interactions have been shown to disrupt replication and transcription culminating in cellular death. Being non-genotoxic, the compounds are less likely to induce second malignancies that is their crucial advantage. Moreover, when being used in combination chemotherapy with genotoxic agents, they allow the reduction of the effective dose of cytostatics.

The variety of DNA-binding small molecules demonstrating antitumor activity includes above 90 natural indolo[2,3-a]carbazoles as well as their synthetic derivatives. This class of heterocyclic compounds is characterized by a plain core consisting of indole and carbazole fragments. Growing interest in the class is attracted by the antibacterial, antimycotic, antiviral, and, especially, anticancer activities of indolo[2,3-a]carbazoles [1,2,3,4].

The studies on indolo[2,3-a]carbazole derivatives show that their antitumor activity is based on the molecular mechanisms of two types. The first one is noncovalent binding to DNA with associated effects: inhibition of topoisomerases, suppression of replication and transcription [5,6,7,8,9,10,11]. This mechanism is typical of rebeccamycin analogs—indolo[2,3-a]carbazoles that include in their structure an imide group and have one bond between the plain fragment and the carbohydrate component. Intercalation is facilitated by a plain indolocarbazole core while the carbohydrate moiety binds the minor or major DNA grooves. Inhibition of topoisomerase is associated with the ability to intercalation: rebeccamycin analogs intercalate between base pairs (that flank one-strand or double-strand breaks induced by topoisomerases) thus preventing the enzyme from ligating DNA. Hence, indolocarbazoles act as ‘topoisomerase poisons’ causing inhibition by interaction with DNA without interaction with the enzyme [12,13].

The second branch is the inhibition of serine/threonine and tyrosine protein kinases that implies direct modulation of the key signal transduction pathways in cancer cells [14,15,16,17,18,19,20,21,22,23,24,25]. This molecular mechanism is mainly realized by staurosporine and its derivatives. This group of indolo[2,3-a]carbazoles is characterized by an amide group in their structure and two bonds between the plain fragment and the carbohydrate component. Staurosporine analogs compete with ATP molecules for the ATP-binding sites of protein kinases: indolocarbazole rings interact with the adenine-binding pocket and the carbohydrate moiety is connected with the ribose-binding site by hydrogen bonds and hydrophobic interactions [26,27,28]. Staurosporine and its derivatives imitate ATP form and its pattern of chemical bonds. It allows them to inhibit a wide range of enzymes—serine/threonine and tyrosine protein kinases as well as ABC-transporter family (P-glycoprotein and ABCG2) which contribute to multiple drug resistance [29,30].

**Figure 1:**
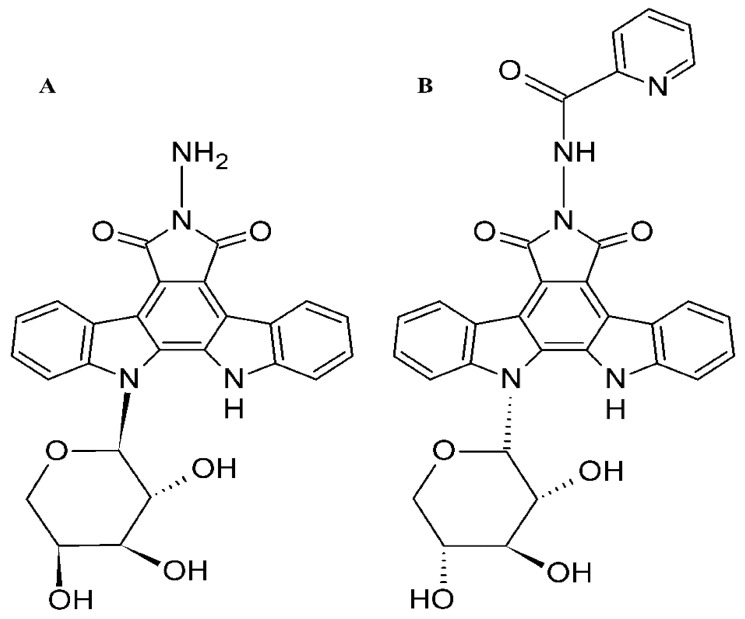
Structural formulae of LCS-1208 (**A**) and LCS-1269 (**B**).

Our research group synthesized a set of indolocarbazole derivatives named LCS that had not been described in the literature before (Figure 1). A number of them were proved to have anticancer activity. The recent research showed that LCS-1208 demonstrated the highest antitumor activity among 10 LCS indolocarbazoles in the following mouse tumor models: P-388 (lymphocytic leukemia), LLC (Lewis lung epidermoid carcinoma) and B-16 (melanoma) [31]. Other study (with 14 compounds of LCS group) revealed that LCS-1269 is the most active in ascitic and 5 solid models. It also demonstrated high anticancer activity in 5 solid models [32].

These two most active compounds—LCS-1208 and LCS-1269—were selected for subsequent research. Their cytotoxic activity in cancer cell lines was estimated by MTT assay. We studied the mechanisms of anticancer activity in accordance with the two aforementioned directions. Firstly, we analyzed LCS-DNA interactions in circular dichroism (circular dichroism in cholesteric liquid crystals) and electrophoretic mobility experiment. In view of LCS-DNA complex formation, the impact on the catalytic activity of topoisomerases I and II was also addressed. As it is known that intercalation and inhibition of topoisomerases induces interferon signaling [33,34], expression of its target genes was also estimated. In addition, chromatin remodeling was analyzed as a probable consequence of LCS-DNA binding—particularly, the effect of the compounds on histone H1 localization and epigenetic control of gene expression.

What is more, the particular item was the analysis of LCS-induced expression changes in a wide gene set. It included genes involved in neoplastic transformation and target genes of the main signaling pathways in cancer cells.

## 2. Materials and Methods

### 2.1. Cell Culture

Cell lines were obtained from Blokhin CRC cell collection. Cells were cultured in DMEM (for adhesion cell cultures) or RPMI-1640 (for suspension cell cultures) supplemented with L-glutamine (0.584 mg/mL), penicillin (50 U/mL) and streptomycin (50 μg/mL) (PanEco, Moscow, Russia) and 10% fetal bovine serum (PanEco, Russia). Cell lines were incubated at 37 °C in 5% CO_2_.

### 2.2. Cell Cytotoxicity Assay

Adhesion and suspension cell lines were seeded in 96-well plates (10,000 cells per well) in 200 μL of DMEM. They were treated with various concentrations of compounds for 72 h. Then 10 μL of MTT-reagent (PanEco, Russia) solution (5 mg/mL; 0.9% NaCl) was added to each well and incubated for 4 h at 37 °C. The medium was removed. Formazan was dissolved in 100 µL of DMSO. The absorbance was recorded at 570 nm by an automated microplate reader Multiscan FC (Thermo Scientific, Waltham, MA, USA). All experiments were performed in parallel and in triplicate.

### 2.3. Treatment, RNA Isolation, cDNA Preparation, and qRT-PCR

The influence of the compounds on the expression of target genes was evaluated in HT29 cancer cell line using qRT-PCR analysis. Cells were incubated for 24 h in the medium with various concentrations of the compounds in 6-well plates. Then the total RNA was extracted with TRIzol reagent (ThermoScientific, USA) according to the manufacturer’s protocol. cDNA was synthesized by a reverse transcription reaction. Total RNA (2 μg, from both control and treated cells) was reverse-transcribed using MMLV-RT reverse transcriptase and random hexamer primers in a 25 μL reaction volume following the manufacturer’s protocol (Syntol, Moscow, Russia). Real-time PCR was conducted in 96-well plates RT^2^ Profiler^TM^ PCR Array Human Cancer PathwayFinder (QIAGEN, Hilden, Germany) and RT^2^ Profiler^TM^ PCR Array Human Signal Transduction PathwayFinder (QIAGEN, Germany). Reaction mixture in each well was prepared according to the manufacturer’s protocol. The thermal cycling conditions were as follows: an initial denaturation step by heating at 95 °C for 10 min, followed by 40 cycles of 15 s initial denaturation (at 95 °C) and 1 min of annealing and extension in 60 °C. The expression of the gene of interest was normalized to the constitutively expressed housekeeping genes (ACTB, B2M, GAPDH, HPRT1, RPLP0). The relative expression level was calculated for each sample using the 2^−ΔΔCt^ method in the manufacturer’s software. All experiments were performed three times and in triplicate.

### 2.4. Circular Dichroism in Cholesteric Liquid Crystals

Circular dichroism (CD) spectra were registered with a spectrometer Chirascan (Applied Photophysics Ltd., UK) in quartz cuvettes (0.4 × 1.0 cm) with an optical path length of 1 cm. Spectra were recorded in the wavelength range 220–600 nm. Salmon sperm DNA was used with molecular weight (0.5–0.8) × 106 Da. Cholesteric liquid crystals (CLC) were prepared by mixing salt DNA solution (0.3 M NaCl, 0.01 M NaH2PO4; pH 6.8) with equal volume of PEG (molecular weight—4000 Da; 340 mg/mL). CD spectra were registered 1 h after mixing. Absorption peak at 285 nm with Δε = 130–140 M^−1^ cm^−1^ was the evidence of CLC formation. In the next step, the compound (1 mM solution in DMSO) was added in small portions (10 μL) with intensive mixing. Then CD spectra were registered in the absorption bands of DNA and ligand (310–330 nm).

### 2.5. Fluorescent Intercalator Displacement Assay (FID)

Concentrations of thiazole orange and oligonucleotide were optimized and equaled 0.5 and 0.25 μM, respectively. The compounds were added in an optimized range of concentrations. Mixture also included buffer for FID (50 mM KCL, 10 mM KH2PO4, 1 mM K2EDTA, pH 7.4). Mixtures were incubated at room temperature for 30 min to establish thermodynamic equilibrium. Every concentration was analyzed in triplicate and every experiment was performed three times. The assay was conducted in 96-well plates. Fluorescence was registered with Spectra Max Plus Microplate Spectrophotometer (Molecular Devices, San Jose, CA, USA) (λex = 513 nm; λem = 533 nm). 

Binding constant was calculated according to the following equation:

Kb = (I(0)/I(L) − 1) ∗ (1 + KfF)/L

where Kb and Kf—compound and thiazole orange binding constants, respectively; 

L and F—compound and thiazole orange concentrations, respectively; 

I(0) and I(L)—thiazole orange fluorescence without compound and in the presence of compound with concentration of L.

Thiazole orange binding constant for DNA duplexes was applied in accordance with the published data: Kf = 3 × 10^6^ M^−1^. DNA duplex sequence was as following: 5′-CAATCGGATCGAATTCGATCCGATTG-3′ [35].

### 2.6. Topoisomerase I Activity Assay

Nuclear extract from MCF-7 cell line including topoisomerase I was incubated with 200 ng of supercoiled pUC19 plasmid DNA and compounds at 37 °C for 30 min. Reaction mixture included topoisomerase I buffer (10 mM tris-HCl, pH 7.9; 1 mM EDTA; 0.15 M NaCl; 0.1% BSA; 0.1 mM spermidine, 5% glycerol) and final volume was 10 μL. Reaction was stopped with the addition of SDS (final concentration of 1%) and proteinase K (final concentration of 50 μg/mL) and subsequent incubation at 55 °C for 1 h. Reaction products were separated by electrophoresis in a 1% agarose gel with TAE-buffer (40 mM tris-base, 1 mM EDTA, 20 mM acetic acid). Electrophoresis was conducted at a voltage of 2 V/cm for 3 h. DNA was stained with EtBr (0.5 μg/mL) and visualized in ultraviolet (240–360 nm).

### 2.7. Topoisomerase II Activity Assay

Human topoisomerase IIα (TopoGEN, Buena Vista, CO, USA) was incubated with 200 ng of supercoiled pHOT-1 plasmid DNA and compounds at 37 °C for 30 min. Reaction mixture included Topoisomerase II Assay Buffer (TopoGEN, Mundelein, IL, USA) and final volume was 20 μL. Reaction was stopped with the addition of SDS (2 μL of 10% solution) and proteinase K (final concentration of 50 μg/mL) and subsequent incubation at 37 °C for 15 min. Reaction products were separated by electrophoresis in a 1% agarose gel with TAE-buffer (40 mM tris base, 1 mM EDTA, 20 mM acetic acid). Electrophoresis was conducted at a voltage of 2 V/cm for 3 h. DNA was stained with EtBr (0.5 μg/mL) and visualized in ultraviolet (240–360 nm).

### 2.8. Live Cell Microscopy

Cells were seeded in 6-well plates (150,000 cells per well) with cover glass 22 × 22 mm for microscopy. After 24 h, cells were incubated with CBL0137 (positive control) or with LCS-1208 and LCS-1269 for 24 h. After treatment, cells were washed with PBS and fixed in 4% paraformaldehyde at room temperature for 10 min. Images were obtained with a Zeiss Axio Observer A1 inverted microscope with N-Achroplan 100×/1.25 oil lens, Zeiss MRC5 camera, and AxioVision Rel.4.8 software. Experiments were performed three times.

### 2.9. Flow Cytometry

Flow cytometry was used to analyze the ability of the drugs to reactivate the expression of epigenetically repressed GFP. Cells were seeded in 24-well plates at 2.5 × 10^4^ cells per well and incubated with LCS-1208 (concentration range 2–0.25 μM) and LCS-1269 (concentration range 4–0.5 μM) for 72 h. The medium in the wells was replaced with fresh one 24 h after treatment and then the cells were incubated for an additional 48 h. Next, the cells were detached from the culture plates using 0.25% trypsin-EDTA solution (PanEco, Russia) and washed with PBS. To maintain high cell viability, PBS solution with 2% fetal bovine serum was used as a cell-storing buffer. The relative number of GFP-positive cells was assessed using BD FACSCanto™ II flow cytometer. Histone deacetylase inhibitor Trichostatin A (TSA) was used as a positive control. Maximum concentration of DMSO for TSA, LCS-1208, and LCS-1269 in media was 0.01%. Experiments were performed three times.

### 2.10. Treatment, Histone Extraction and Western Blot

The effect of LCS-1269 on histone modifications was assessed on HeLa TI cells using Western blotting. Cells were incubated with LCS-1269 at concentrations of 2 and 4 μM for 8, 24 and 72 h in 60 mm Petri dishes. The histone fraction of proteins was obtained using the Abcam acid extraction protocol and trichloroacetic acid precipitation as previously described [36]. Histone proteins were separated by 15% PAGE and transferred to 0.22 μm nitrocellulose membranes for 40 min at 100 mA; membrane blocking was performed using a 2.5% BSA solution in PBST for 30 min. Membranes were incubated with the following primary antibodies at 4 °C overnight: poly-H3ac (sites K9, K14, K18, K23, K27) (ab47915), H3K9me3 (ab8898), H4K20me3 (ab9053) and H4 as loading control (ab10158). Incubation with anti-rabbit (ab6721) and anti-mouse (ab6728) secondary antibodies was carried out at 4 °C for 2.5 h. Proteins were detected using Clarity Western ECL Substrate visualization reagent (1705061, Bio-Rad, Hercules, CA, USA) and ImageQuant LAS 4000 digital imaging system (GE Healthcare, Chicago, IL, USA). Densitometric analysis of the blots was performed using ImageJ software. Experiments were performed three times.

### 2.11. Treatment, Nuclear Extraction and Western Blot

The effect of LCS-1269 on HDAC expression was assessed on HeLa TI cells using Western blotting. Cells were incubated with LCS-1269 at concentrations of 2 and 4 μM for 24 h in 60 mm Petri dishes. The nuclear fraction of the cells was obtained according to the Abcam Nuclear extraction and fractionation protocol. Nuclear proteins were separated by 10% PAGE and transferred to 0.22 μm nitrocellulose membranes; transfer conditions for proteins up to 25 kDa were 40 min at 100 mA, for proteins from 25 to 100 kDa—40 min at 100 mA and 20 min at 200 mA. Blocking with BSA, primary and secondary antibodies was performed as described above. The study used Abcam antibodies to HDAC1 (ab53091), histone H4 (load control, ab10158) and secondary anti-rabbit antibodies (ab6721). Protein detection and densitometric analysis were performed as described above. Experiments were performed three times.

### 2.12. Treatment, DNA-Isolation, DNA Methylation ELISA and Methylation-Sensitive HpaII/MspI Restriction Enzyme Assay (MSRE)

The effect of LCS-1269 on integral DNA methylation was analyzed on CasKi cells using two methods: DNA Methylation ELISA and methylation sensitive HpaII/MspI restriction assay. Cells were seeded in 6-well plates (1.5 × 10^5^ cells per well) and incubated with 4 nM of compound for 72 h. After every 24 h of incubation, half of the culture medium was replaced with fresh medium and the drug was added to the original concentration. Genomic DNA was extracted from the cells using the GeneJET Genomic DNA Purification Kit (K0721, Thermo Scientific, Mundelein, IL, USA). First, the level of global DNA methylation after LCS-1269 treatment was performed using The MethylFlash™ Global DNA Methylation (5-mC) ELISA Easy Kit (P-1030-96, EpiGentek, Farmingdale, NY, USA) according to manufacturer’s protocol. The enzymatic reaction with HpaII and MspI restriction enzymes was performed using EpiJet kit (K1441, Thermo Scientific, Mundelein, IL, USA) according to the manufacturer’s protocol. Restriction products were analyzed by 1% agarose gel electrophoresis with TAE-buffer and detected on a Typhoon 9400 scanner (GE Healthcare, Chicago, IL, USA). All experiments were performed three times.

### 2.13. qRT-PCR for Interferon Signaling and Gene Expression of Histone Methyltransferases, Histone Deacetylases and DNA Methyltransferases

The effect of the compounds on interferon signaling and gene expression of histone methyltransferases, histone deacetylases and DNA methyltransferases was studied by means of qRT-PCR analysis. The influence of the compounds was evaluated in HeLa cancer cell line. Cells were incubated for 24 h in the medium with various concentrations of the compounds in 6-well plates. Then the total RNA was extracted with TRIzol reagent (Thermo Scientific, Mundelein, IL, USA) according to the manufacturer’s protocol. cDNA was synthesized by a reverse transcription reaction. Total RNA (1 μg, from both control and treated) was reverse-transcribed using MMLV-RT reverse transcriptase and random hexamer primers in a 25 μL reaction volume following the manufacturer’s protocol (Syntol, Russia). Real-time PCR was conducted in reaction mixture according to manufacturer’s protocol (Syntol, Russia). The thermal cycling conditions were as follows: an initial denaturation step by heating at 95 °C for 5 min, followed by 40 cycles of 15 s initial denaturation (at 95 °C), 20 s at appropriate melting temperature according to the primers and 25 s of extension in 72 °C. The expression of the gene of interest was normalized against the constitutively expressed housekeeping gene RPL27. The relative expression level was calculated for each sample using the 2^−ΔΔCt^ method. All experiments were performed three times and in triplicate.

The sequences of primers used for qRT-PCR were as listed in Table 1.

### 2.14. Statistical Methods

To assess the significance of differences between the groups, including the expression levels, a paired two-sample Student’s *t*-test with a level of statistical significance of *p* < 0.05 was used.

## 3. Results

### 3.1. Cytotoxicity of LCS-1208 and LCS-1269 in Human Cancer Cell Lines

Cytotoxicity was analyzed in 8 adhesion and 4 suspension human cell lines. Cytotoxicity level is presented in Table 2.

LCS-1269 demonstrated cytotoxicity with IC_50_ in the range of 1.2 to 31 µM. Minimal cytotoxicity was observed in breast adenocarcinoma cell line MCF-7 and immortalized keratinocytes HaCaT (31 and 29 µM, respectively); maximal cytotoxicity was detected for glioblastoma cell line U251 (1.2 µM).

LCS-1208 demonstrated the IC_50_ range of 0.13 to 31 µM. Minimal LCS-1208 cytotoxicity was found in HaCaT cell line (31 µM); maximal one—in colorectal adenocarcinoma cells HT29 (0.13 µM).

LCS-1208 showed higher cytotoxicity than LCS-1269 in all cell lines. Both compounds demonstrated the highest cytotoxicity level in HT29 and U251. The lowest level was registered in HaCaT. Thus, LCS-1208 and LCS-1269 are more active against tumorigenic cancer cell lines than immortalized cells.

Among the suspension cell lines, LCS-1269 showed minimal cytotoxicity in chronic myelogenous leukemia cells K562; the maximum of cytotoxicity—in B-cell lymphoma cell line Granta-519. LCS-1208 demonstrated the activity with IC_50_ in the range of 0.071 to 6 μM. The highest level was detected in Granta-519, the lowest level—in K562.

Cytotoxicity of LCS-1208 was higher than the activity of LCS-1269 in all suspension cell lines. IC_50_ for K562 under LCS-1269 treatment was not detected as it exceeds the range of solubility.

### 3.2. Affinity of LCS-1208 and LCS-1269 to DNA Duplex

To determine the possible intracellular targets of the compounds, we firstly analyzed the effects on the most probable one—DNA molecules. The affinity of polyphenols to DNA duplexes was measured by means of FID assay [37]. The method is based on fluorophore (thiazole orange) displacement from an oligonucleotide by a competing molecules (ligands). Displacement leads to fluorescence quenching. The affinity of compounds is characterized by a binding constant which is derived from the fluorescence intensity-concentration curve.

Binding constant of LCS-1208 equals (1.24 ± 0.07) × 10^6^ M^−1^. LCS-1269 demonstrated lower affinity with binding constant (2.52 ± 0.4) × 10^5^ M^−1^. Therefore, both compounds bind DNA that is typical of rebeccamycin derivatives.

### 3.3. Analysis of LCS-DNA Complexes by Means of Circular Dichroism in Cholesteric Liquid Crystals

We also analyzed the mechanism of interaction between LCSs and DNA by means of circular dichroism in cholesteric liquid crystals (CD in CLC). CD in CLC was previously described [38,39,40].

It is a crucial moment that CD spectra of compounds, that include chromophores and bind DNA, allowing the estimation of the angle between the chromophore and helical axis of DNA. If the angle is in the range of 54–90°—the CD band has a minus sign. If the angle is in the range of 0–54°—the CD band has a plus sign. Thus, the intercalation (α ~ 90°) can be detected by registration of a negative peak in the absorption wavelength of the compound. 

For instance, being chromophores nitrogen bases of DNA demonstrate a negative peak in the absorbance band of DNA. Negative peak is registered due to the angle between nitrogen bases and helical axis of DNA being about 90°.

**Figure 2:**
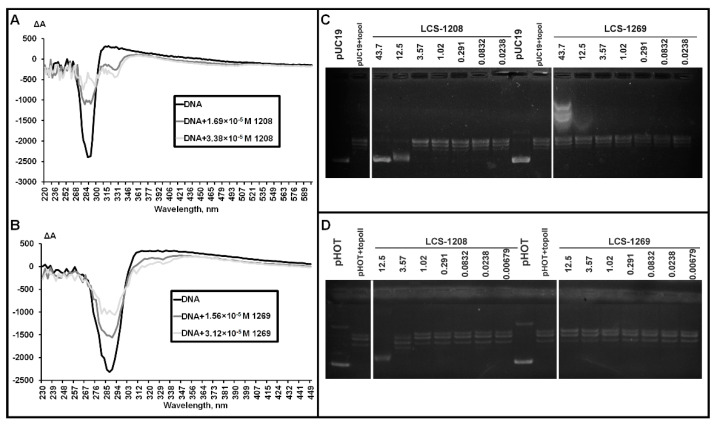
(**A**,**B**). CD spectra of CLC of DNA without compounds and in the presence of: (**A**) LCS-1208; (**B**) LCS-1269. (**C**) Distribution of reaction products visualized after electrophoresis in 1% agarose gel. Line pUC19—supercoiled plasmid DNA; pUC19 + topoI—products of relaxation by topoisomerase I. Lines with concentrations—products of relaxation by topoisomerase I in the presence of LCS-1208 and LCS-1269 in respective concentrations (μM). (**D**) Distribution of reaction products visualized after electrophoresis in 1% agarose gel. Line pHOT—supercoiled plasmid DNA; pHOT + topoII—products of relaxation by topoisomerase IIα. Lines with concentrations—products of relaxation by topoisomerase IIα in the presence of LCS-1208 and LCS-1269 in respective concentrations (μM).

CD spectra in the presence of LCS-1208 and LCS-1269 are presented in Figure 2A,B.

LCS-1208 is detected by a negative peak in the absorbance band of the compound (325 nm). Minus sign of the peak means that the angle between the chromophore plane and the helical axis of DNA is in the range of 54–90°. This range is typical of intercalation complexes [39].

Significant decrease of DNA peak indicates a partial disruption of CLC which can be the consequence of intercalation.

CD spectrum in the presence of LCS-1269 demonstrates a decrease of the DNA peak. It is the evidence of LCS-1269 binding to DNA. However, the type of complex cannot be unambiguously defined because of the low peak of LCS-1269. Its minus sign may indicate intercalation with the angle being about 54° [40]. This type of intercalation is presumably caused by large substituents in the chromophore. 

Low amplitude may also be explained by multiple mechanisms of interaction. For instance, simultaneous binding by intercalation mode, groove binding, and external binding—in this case bands with opposite signs become superimposed.

### 3.4. Topoisomerase I Assay

As LCS-1208 and LCS-1269 are derivatives of rebeccamycin, their anticancer and cytotoxic effects are expected to be based on intercalation into DNA molecules and inhibition of topoisomerases. The effect of the compounds on topoisomerase I activity was studied in the reaction of plasmid relaxation. Products of the reaction were separated in agarose gel by means of electrophoresis. Relaxation leads to a decrease of mobility. As a result of incomplete relaxation, topoisomers can also be observed as additional lines in close proximity to the relaxed form (Figure 2C).

LCS-1208 inhibited the enzyme in a concentration of 43.7 μM. The compound also partially inhibited topoisomerase I in a concentration of 12.5 μM. Lower concentrations did not cause any suppression of catalytic activity. Thus, LCS-1208 inhibits the enzyme in a dose-dependent manner.

LCS-1269 did not show any effect on topoisomerase I activity.

### 3.5. Topoisomerase IIα Assay

To study the effect of LCS-1208 and LCS-1269 on the activity of topoisomerase IIα, we performed the reaction of plasmid relaxation by the purified enzyme. Products of the reaction were separated in agarose gel by means of electrophoresis (Figure 2D).

LCS-1208 inhibits topoisomerase IIα in a dose dependent manner. Concentration of 3.57 µM is associated with partial inhibition and maximal concentration (12.5 µM)—with full inhibition.

The experiment with LCS-1269 in the same range of concentrations did not reveal inhibitory activity.

### 3.6. The Effect of LCS-1208 and LCS-1269 on Interferon Signaling

Recent studies show that intercalators and topoisomerase IIα inhibitors activate interferon signaling due to the induction of DNA breaks. We analyzed the influence of the compounds on the activity of interferon pathways. By means of qRT-PCR, we detected the effects of the compounds on the expression of interferon-induced genes in HeLa cell line.

**Figure 3:**
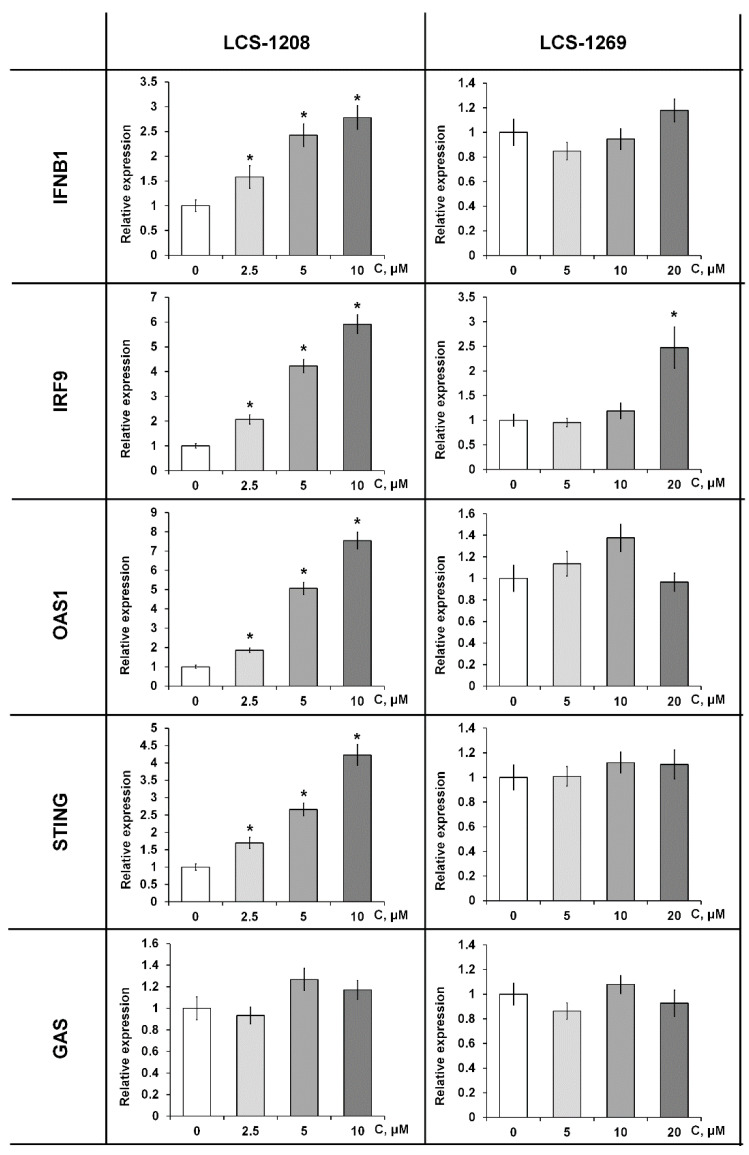
Relative expression of interferon-induced genes in HeLa cell line after treatment with LCS-1208 and LCS-1269 for 24 h. *p* values were determined using *t*-test. All data are presented as M ± SD. *—differences are statistically significant compared to the control (*p* < 0.05).

LCS-1208 upregulates expression of interferon-induced genes (IFNB1, IFN9, OAS1, and STING) in a dose-dependent manner (Figure 3).

The influence of LCS-1269 was not registered (Figure 3). Increase of expression was observed for IRF9 in maximal concentration.

### 3.7. Chromatin Remodeling

#### 3.7.1. Linker Histone H1 Displacement

DNA-binding compounds are able to remodel chromatin due to displacement of the linker and core histones. We studied the effect of LCS-1208 and LCS-1269 on histone H1 localization by means of live cell microscopy in HeLa cell culture with mCherry-tagged H1 (HeLa-H1-mCherry).

**Figure 4:**
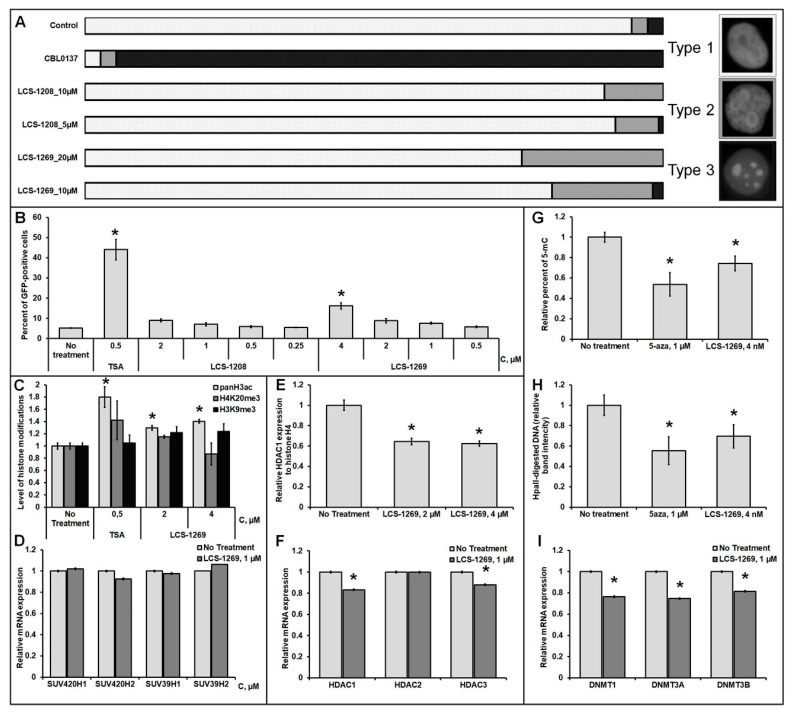
(**A**). Displacement of histone H1 from chromatin in HeLa-H1-mCherry cells after treatment with LCS-1208 and LCS-1269 for 24 h; types 1,2,3—different types of histone H1 localization in nucleus. (**B**). Analysis of the epigenetic activity of LCS-1208 and LCS-1269 in HeLa TI cell-based assay, flow cytometry results. (**C**). Analysis of the LCS-1269 effects on histone modifications, Western blot results. (**D**). Analysis of the LCS-1269 effects on gene expression of the histone methyltransferases, qRT-PCR results. (**E**,**F**). Analysis of the LCS-1269 effects on HDACs expression, Western blot, and RT-PCR results. (**G**–**I**). Analysis of the LCS-1269 effects on DNA methylation, ELISA, MSRE assay, and qRT-PCR results. All data are presented as M ± SD. *—differences are statistically significant as compared to the control (*p* < 0.05).

CBL0137 (as a positive control) relocalized H1 into nucleoli as it intercalates into DNA molecules and displaces linker histones from chromatin to the nucleoplasmic fraction (Figure 4A). LCS-1208 did not induce statistically significant relocalization of H1. On the other hand, LCS-1269 displaces histone H1 with statistical significance (*p* < 0.05) after treatment with concentrations of 20 and 10 µM in 25% and 19% of cells, respectively.

#### 3.7.2. Analysis of the LCS-1208 and LCS-1269 Ability to Reactivate the Expression of the Epigenetically Repressed GFP Gene in HeLa TI Cell-Based Assay

Our analysis of chromatin remodeling also included a wide set of epigenetic experiments. To determine the epigenetic activity of LCS-1208 and LCS-1269, the HeLa TI test system was used. HeLa TI is a polyclonal population of HeLa cells containing a genome-integrated vector (derived from avian sarcoma retrovirus) carrying epigenetically repressed GFP and capable of detecting inhibitors of histone deacetylases, DNA methyltransferases, bromodomains and some histone methyltransferases [41,42]. The baseline level of GFP-positive cells in the population ranges from 3.5 to 5%. Epigenetic reactivation of the GFP gene is realized by an increase in the number of cells with a GFP-positive phenotype. The results of flow cytometry showed that when the cells were treated with the compound LCS-1269, there was a dose-dependent increase in the number of GFP-positive cells, which reached a 6-fold increase in the maximum concentration. Under the action of LCS-1208, a weak reactivation of the GFP gene was revealed (Figure 4B). For all concentrations used, cell survival did not decrease less than 90%. Thus, the LCS-1269 substance was identified as a potential epigenetic modulator; therefore, we further focused on studying the effects of LCS-1269 on histone modifications and DNA methylation.

#### 3.7.3. Analysis of LCS-1269 Effect on Histone Modifications

Analysis of the effect of LCS-1269 on histone modifications was carried out at several levels. In the first part of the study, we evaluated the effect of the agent on the change in the level of histone modifications associated with actively transcribed chromatin (acetylation of histone H3 in sites K9, K14, K18, K27) and modifications associated with transcription repression (H3K9me3, H4K20me3, H3K27me3) by the method of Western blot. The experimental results showed that under the action of LCS-1269, there is a small but statistically significant increase in histone H3 acetylation (1.2 times) (Figure 4C and Figure S1). At the same time, no changes in the level of other modifications under the action of LCS-1269 were observed. In addition, we assessed the level of expression of genes encoding histone methyltransferases responsible for the modifications H3K9me3 (*SUV39H1, SUV39H2*) and H4K20me3 (*SUV420H1, SUV420H2*) (Figure 4D). Thus, the data obtained demonstrate that LCS-1269 does not affect both the level of the modifications themselves and the level of expression of the genes of the histone methyltransferases associated with them. Acetylation of histone H3 is mainly regulated by enzymes from the class of histone deacetylases (HDACs), therefore, at the next stage, we analyzed the ability of the compound to inhibit the expression of the HDAC1 protein and mRNA genes *HDAC1, HDAC2, HDAC3.* It was shown that LCS-1269 caused a decrease in HDAC1 expression at both the mRNA (1.2 times) and protein levels (1.6 times) (Figure 4E,F and Figure S2). Additionally, a slight decrease in expression of the HDAC3 gene was shown (Figure 4F).

#### 3.7.4. Analysis of LCS-1269 Effect on DNA Methylation

To analyze the effect of the agent on integral DNA methylation, we used 2 methods: methyl-sensitive restriction analysis and DNA Methylation ELISA. Restriction analysis is based on the action of the enzymes MspI and HpaII, the former of which recognizes C ^ CGG sites and cleaves them independently of their methylation, while HpaII recognizes and cleaves only unmethylated C ^ CGG sites. Thus, the demethylating activity of the compounds can be determined based on the ratio of DNA restriction products to undigested DNA from treated and untreated cells. The second method involves the interaction of specific antibodies with methylated cytosine (5-μC) of total DNA, followed by enzymatic detection. DNA Methylation ELISA allows one to assess the effect of a compound on global DNA methylation and to determine the total percentage of methylated cytosines in DNA. The data obtained with both methods showed that there is a 20% decrease in the degree of DNA methylation under the action of LCS-1269 (Figure 4G,H and Figure S3). In addition, DNA methyltransferase gene expression analysis was performed, which showed a 1.3-fold decrease in the expression level of *DNMT1* and *DNMT3A* after treatment of cells with the compound (Figure 4I).

### 3.8. The Analysis of LCS-Induced Expression Changes in a Gene Set of Neoplastic Transformation

We also suggested that the antitumor and cytotoxic activities of the compounds are based not only on mechanisms typical of rebeccamycin analogs (intercalation and topoisomerase inhibition) but on alternative ones such as alterations in signaling pathways. The latter mechanism is also widespread among indolocarbazoles. 

96-well plates RT^2^ Profiler^TM^ PCR Array Human Cancer PathwayFinder (QIAGEN, Germany) were used in the experiment. This kit includes primers for 84 genes involved in: regulation of cell cycle; apoptosis; DNA repair and response to DNA damage; angiogenesis; epithelial-mesenchymal transition; regulation of telomerase activity and telomere maintenance; cellular senescence; metabolism; response to hypoxia. Malfunction of these genes contributes to neoplastic transformation.

Treatment with LCS-1208 resulted in the change of expression in 6 genes (Table S1). 2 genes were downregulated and 4—upregulated. LCS-1208 modulated the following clusters: angiogenesis (FLT1), apoptosis (BCL2L11, BIRC3, CASP7), cellular senescence (IGFBP3, IGFBP7).

LCS-1269 modulated expression of 9 genes (Table S1). 8 genes demonstrated reduction of expression and 1 gene was upregulated. These 9 genes are in the following clusters: angiogenesis (KDR, VEGFC), apoptosis (BIRC3, FASLG), cell cycle (SKP2), cellular senescence (IGFBP3), DNA repair and response to DNA damage (GADD45G), epithelial-mesenchymal transition (KRT14), response to hypoxia (EPO).

### 3.9. The Analysis of LCS-Induced Expression Changes in a Set of Target Genes of Signal Pathways

96-well plates RT^2^ Profiler^TM^ PCR Array Human Signal Transduction Pathway Finder (QIAGEN, Germany) were used in the experiment. This kit includes primers for 84 target genes of signal pathways.

Incubation with LCS-1208 led to the change of expression of 15 genes (Table S1). 4 genes were downregulated and 11—upregulated. LCS-1208 modulated the following signal pathways: PPAR (OLR1, SORBS1), Wnt (AXIN2, MMP7, CCND1), NFκB (CSF1, CCL5, ICAM1, TNF), JAK/STAT (CCND1, MCL1), TGFß (GADD45B), Notch pathway (JAG1), Hedgehog pathway (BMP4) and signaling associated with response to hypoxia (CA9, LDHA).

Change of expression after treatment with LCS-1269 was registered in 8 genes: 5 of them were downregulated and 3 were upregulated (Table S1). These genes are the targets of PPAR signal pathway (SORBS1), TGFß pathway (CDKN1B), NFκB (CCL5), JAK/STAT (CCND1) and Wnt (AXIN2, CCND1), Hedgehog (BMP4), Notch (LFNG) and signaling associated with response to hypoxia (LDHA).

## 4. Discussion

Our study was focused on the cytotoxic activity and molecular mechanisms of anticancer activity of LCS-1208 and LCS-1269 (Table 3). These indolocarbazoles demonstrated the highest antitumor activity in LCS group on mouse tumor models in vivo. 

MTT assay on adhesion cell lines revealed the highest cytotoxicity level in HT29 and U251. In this view, HT29 cell line was selected for subsequent studies on signaling pathways. The lowest level was registered in HaCaT. Thus, LCS-1208 and LCS-1269 are more active against tumorigenic cancer cell lines than immortalized cells. Analysis of the published data concerning gold standard treatment for our cell lines show that, in many cases, our compounds demonstrate comparable effects. For some lines, cytotoxic activity is even superior to that of the gold standard. The data are presented in Table S2. It is also noteworthy that the difference in cytotoxicity level varies between cell lines. While IC_50_ for HepG2 and A549 were within one order, IC_50_ of LCS-1269 in MCF-7, HT29, and PC-3 exceeds IC_50_ of LCS-1208 by 4.8, 10.8 and 24.7 times, respectively. This difference can be explained by the compounds having several intracellular targets. On the other hand, a similar pattern of cytotoxic activity between the two compounds (specificity of cytotoxicity in relation to the cell line) may be determined by the cell membrane permeability of certain cell lines.

To determine these targets, we firstly analyzed the effects on the most probable one—DNA molecules. LCS-1208 and LCS-1269 demonstrated affinity to DNA duplexes in FID experiments. Binding constant of LCS-1208 was higher than that of LCS-1269 and was close to the results of the other group [43]. CD experiment revealed that LCS-1208 binds DNA by intercalating into molecules. However, the conditions required for CLC formation sharply differ from the physiological ones. In view of this fact, we also analyzed LCS-DNA interactions by means of electrophoresis (data not provided). Its results were consistent with CD data. LCS-1208 demonstrated binding to plasmid DNA causing a decrease of electrophoretic mobility. LCS-1269 did not show any change in electrophoretic mobility. This difference can be determined by the substituent in the imide nitrogen atom: the amino group of LCS-1208 facilitates intercalation due to its positive charge [44] while the large picolinamide substituent of LCS-1269 prevents it from intercalating between nitrogenous bases.

LCS-1208 and LCS-1269 are derivatives of rebeccamycin. Anticancer and cytotoxic effects of this group are mainly based on intercalation into DNA molecules and inhibition of topoisomerases [4]. Hence, we analyzed their effects on human topoisomerases I and IIα. LCS-1208 inhibited topoisomerase I and topoisomerase IIα. Its effect on topoisomerase I is consistent with the study of the other group in which it is demonstrated that the enzyme is inhibited in a dose-dependent manner [43]. Inhibition of topoisomerase IIα by LCS-1208 was firstly revealed in our study. LCS-1269 did not demonstrate any influence.

Recent studies show that intercalators and topoisomerase IIα inhibitors activate interferon signaling due to induction of DNA breaks [33,34]. In this study, we demonstrated that LCS-1208 upregulates the expression of interferon-induced genes, while the influence of LCS-1269 is relatively low. These data are consistent with the results of CD and topoisomerase IIα inhibition experiments. Being a strong intercalator and topoisomerase IIα inhibitor, LCS-1208 may induce double-strand breaks with subsequent activation of type I interferon signaling via cGAS-STING cascade [33,34,45]. This pathway is involved in the response to infection as it is activated by bacterial and viral dsDNA [46]. Indeed, dose-dependent upregulation of IFN-α/IFN-β signaling target genes (OAS1, IFNB1, and STING) indicates activation of the type I interferon pathway.

DNA-binding compounds—intercalators as well as minor groove binders—are able to destabilize chromatin due to displacement of the linker and core histones from chromatin [47,48]. We studied the effect of LCS-1208 and LCS-1269 on histone H1 localization by means of live cell microscopy in HeLa cell culture with tagged H1 (HeLa-H1-mCherry). LCS-1208 did not cause relocalization of H1 despite being an intercalator according to CD experiments. It can indicate that LCS-1208 does not alter DNA topology. It is known that some intercalators—perpendicular intercalators (their long axis orients perpendicular to the flanking base pairs)—do not cause DNA unwinding, so LCS-1208 can be from this class [49]. On the other hand, LCS-1269 displaces histone H1 after treatment with both concentrations. According to CD in CLC LCS-1269 binds DNA. However, the type of complex cannot be unambiguously defined. LCS-1208 is a weak intercalator or simultaneously binds by intercalation mode, groove binding and external binding. We have already shown that intercalators and minor groove binders displace H1 from chromatin [50]. Thus, both types of interaction (intercalation as well as groove binding) are able to result in H1 relocalization. As histone H1 interacts with DNA minor groove [51] its displacement by LCS-1269 may indicate that this compound binds the minor grooves of DNA molecules.

The study of chromatin remodeling also included a wide set of epigenetic experiments. They revealed that strong epigenetic modulation (detected in HeLa TI cell-based assay) is provided by a combination of weak effects on H3 histone acetylation (via a decrease in HDAC1 and HDAC3 expression) and total DNA methylation (decrease in the degree of DNA methylation and the expression level of *DNMT1* and *DNMT3A*). It is noteworthy that overexpression of HDACs, in most cases, is associated with advanced disease and poor survival in cancer patients. Several inhibitors of HDACs are used in cancer treatment. In this view, downregulation of HDAC1 and HDAC3 expression is an important advantage of LCS-1269 and can contribute into its anticancer activity [52]. The influence of DNA methylation is more ambiguous. However, promoters of tumor suppressor genes are shown to be hypermethylated in cancer cells. Thus, for instance, tumor cells treated with 5-azaC (a compound that causes hypomethylation of DNA) lose many of their cancer-like properties [53]. Therefore, decrease in the level of DNA methylation facilitates the antitumor effect of LCS-1269.

As LCS-1269 shows weak binding with DNA and associated effects, we suggest that the antitumor and cytotoxic activities of LCS-1269 are based not on mechanisms typical of rebeccamycin analogs (intercalation and topoisomerase inhibition) but on alternative ones such as alterations in signaling pathways.

PCR array with a gene set of neoplastic transformation revealed some patterns. Upregulation of BCL2L11 and CASP7 (proapoptotic genes) caused by LCS-1208 can be associated with multiple DNA breaks. We showed that LCS-1208 inhibits human topoisomerases I and IIα. It is known that inhibition of topoisomerases leads to double-strand breaks in human cells irrelevantly to the mechanism of inhibition (in the case of ‘topoisomerase poisons’ as well as ‘catalytic inhibitors’) [54]. Double-strand breaks induce cell cycle arrest and activate DNA repair systems. Critical number of multiple DNA breaks results in cell death.

LCS-1269 reduced the expression of genes controlling angiogenesis and lymphangiogenesis. Positive regulators of angiogenesis VEGFC and KDR (VEGFR2) demonstrated decrease of expression. For instance, VEGFC is known to initiate lymphangiogenesis. Its product binds VEGFR-3 receptor stimulating proliferation, survival, and migration of lymphatic endothelial cells. What is more, this factor increases the permeability of the endothelial barrier in lymphatic vessels. These functions facilitate the metastasis of tumors, including colorectal cancer [55]. Thus, the reduction of VEGFC expression in the colorectal cancer cell line HT29 is the evidence of a potential antiangiogenic and antimetastatic effects of LCS-1269.

Concerning the analysis of LCS effects on signaling target gene expression, it is noteworthy that LCS-1208 and LCS-1269 downregulate CCND1 expression. This gene encodes cyclin D1 and is a target of JAK/STAT, Wnt, PI3K, and MAP-kinase pathways. Since CCND1 is a positive regulator of cell proliferation, downregulation of its expression can lead to cytostatic effect. LCS-1208 and LCS-1269 also downregulate AXIN2—another target gene of Wnt pathway [56]. This cascade is characterized by malfunction in most cases of colorectal adenocarcinoma [57].

## 5. Conclusions

Revealed molecular mechanisms clarify the base of the anticancer effect of LCS-1208 and LCS-1269. Despite of being structurally similar, each of these compounds has a unique range of biological activities. The effects of LCS-1208 are mainly centered around intercalation and associated mechanisms (inhibition of topoisomerases, induction of interferon signaling, and activation of proapoptotic gene expression). The mechanisms of LCS-1269 antitumor activity are less typical of rebeccamycin derivatives. They include influences on higher levels such as chromatin remodeling and epigenetic effects as well as a few alterations in signal pathways. These nonstandard (for indolocarbazole class) mechanisms of LCS-1269 anticancer activity require further studies.

## Figures and Tables

**Table 1:** Primers for interferon-induced genes and genes of histone methyltransferases, histone deacetylases and DNA methyltransferases.

	Forward Primer	Reverse Primer
RPL27	5′-ACCGCTACCCCCGCAAAGTG-3′	5′-CCCGTCGGGCCTTGCGTTTA-3′
IFNB1	5′-ACGCCGCATTGACCATCTAT-3′	5′-GTCTCATTCCAGCCAGTGCT-3′
IRF9	5′-CAACTGAGGCCCCCTTTCAA-3′	5′-CGCCCGTTGTAGATGAAGGT-3′
OAS1	5′-TGGAGACCCAAAGGGTTGGA-3′	5′-AGGAAGCAGGAGGTCTCACC-3′
GAS	5′-ACGTGCTGTGAAAACAAAGAAG-3′	5′-GTCCCACTGACTGTCTTGAGG-3′
STING	5′-ATATCTGCGGCTGATCCTGC-3′	5′-GGTCTGCTGGGGCAGTTTAT-3′
HDAC1	5′-CACCCATTCTTCCCGTTCTT-3′	5′-GGCATTTCAGGAGTTTGTCTTAT-3′
HDAC2	5′-CTTATTGTGTGTCTGCCCATTT-3′	5′-ATTTGTCTGCTTCCTGCTACT-3′
HDAC3	5′-AATGCCTTCAACGTAGGCGA-3′	5′-GGGTTGCTCCTTGCAGAGAT-3′
DNMT1	5′-AGCACAGAAGTCAACCCAAA-3′	5′-TGCGTCTCTTCTCCTCCTTT-3′
DNMT3A	5′-AGCCCAAGGTCAAGGAGATT-3′	5′-TACGCACACTCCAGAAAGC-3′
DNMT3B	5′-CAACAGCATCGGCAGGAA-3′	5′-GTCCTCTGTGTCGTCTGTGA-3′
SUV420H1	5′-CCCGTGTAGCATAAAAGCAGC-3′	5′-CCAGTTTCACCAAGGAACCAG-3′
SUV420H2	5′-CGTGCTTGGAAGAAGAATGA-3′	5′-GCAGTCATGGTTGATGAAGG-3′
SUV39H1	5′-GCTAGGCCCGAATGTCGTTA-3′	5′-TAGAGATACCGAGGGCAGGG-3′
SUV39H2	5′-GCAGGACGAACTCAACAGAA-3′	5′-CAACCAAAGGTGGCTTCATT-3′

**Table 2:** IC_50_ of LCS-1028 and LCS-1269 in 11 human cell lines (µM). All data are presented as M ± SD.

	LCS-1269	LCS-1208	Histogenesis/Cancer Type
Adhesion lines			
HeLa	26.6 ± 2.1	28.1 ± 1.8	Cervical adenocarcinoma
MCF-7	31 ± 1	5.5 ± 0.7	Breast adenocarcinoma
HepG2	2.5 ± 0.5	1.7 ± 0.3	Hepatocellular carcinoma
U251	1.2 ± 0.06	0.36 ± 0.08	Glioblastoma
A549	3.2 ± 0.3	1.0 ± 0.01	Lung adenocarcinoma
PC-3	24 ± 4	0.97 ± 0.01	Prostatic adenocarcinoma
HT29	1.4 ± 0.5	0.13 ± 0.01	Colorectal adenocarcinoma
HaCaT	29 ± 2	31 ± 3	Spontaneously immortalized keratinocytes
Suspension lines			
CCRF CEM	6.8 ± 0.7	2.0 ± 1.2	Acute lymphoblastic leukemia
K562	>500	6.0 ± 0.06	Chronic myelogenous leukemia
KG-1	7.1 ± 2.3	0.6 ± 0.2	Acute myelogenous leukemia
Granta-519	0.60 ± 0.02	0.071 ± 0.008	B-cell lymphoma

**Table 3:** The effects of LCS-1208 and LCS-1269 in the study.

			LCS-1208	LCS-1269
**Interaction with DNA**		**Mechanism of interaction (CD in CLC)**	Intercalation	Intercalation or groove binding
		**Binding constant (FID)**	1.24 × 10^6^ M^−1^	2.52 × 10^5^ M^−1^
		**Decrease of DNA electrophoretic mobility (Electrophoresis)**	+	-
**Inhibition of topoisomerase I**			+	-
**Inhibition of topoisomerase IIα**			+	-
**Activation of interferon signaling**			+	-
**Displacement of histone H1**			-	+
**Influence on the following mechanisms of epigenetic regulation of gene expression:**	**Expression of epigenetically repressed GFP gene (HeLa TI cell-based assay)**		-	Reactivation
	**DNA methylation**			Decrease
	**DNA methyltransferase gene expression**			Decrease
	**Histone modifications associated with actively transcribed chromatin and repressed transcription**			Increase in H3 acetylation (actively transcribed chromatin)
	**Expression of genes of histone deacetylases**			Decrease in HDAC1 and HDAC3 expression
	**Expression of genes encoding histone methyltransferases**			-

## Data Availability

The data presented in this study are available on request from the corresponding author.

## Supplementary Materials

The following are available online (PDF). Table S1: Relative expression of the genes in HT29 cell line after treatment with LCS-1208 and LCS-1269 for 24 h, Table S2: The data on cytotoxicity of gold standard treatment compounds and LCSs in 11 tumor cell lines, Figure S1: Analysis of the LCS-1269 effects on histone modifications, Western blot results. Figure S2: Analysis of the LCS-1269 effects on HDACs expression, Western blot results. Figure S3: Analysis of the LCS-1269 effects on DNA methylation MSRE assay results.

## Abbreviations

MTT, (3-(4,5-dimethylthiazol-2-yl)-2,5-diphenyl tetrazolium bromide); FID, fluorescent intercalator displacement; CD in CLC (circular dichroism in cholesteric liquid crystals).
