# Supplementary material for: Molecular Mechanisms of Anticancer Activity of N-Glycosides of Indolocarbazoles LCS-1208 and LCS-1269
Source: Molecules. 2021 Dec 2;26(23):7329. doi: 10.3390/molecules26237329 (PMC8658795; doi:10.3390/molecules26237329)
Supplement: Supplementary file 1 [file molecules-26-07329-s001.zip › molecules-1468930-supplementary.pdf]

Supplementary materials

Molecular mechanisms of anticancer activity of N-glycosides of indolocarbazoles LSC-1208 and LSC-1269

Roman G. Zenkov*[1], Lidiya V. Ektova[1], Vera A. Eremina[1], Olga A. Vlasova[1], Varvara P. Maksimova[1], Timur I. Fetisov[1], Natalia Y. Karpechenko[1], Valeriia G. Popova[1,4], Olga G. Usalka[1,3], Ekaterina A. Lesovaya[1,5], Gennady A. Belitskiy[1], Marianna G. Yakubovskaya[1], Kirill I. Kirsanov[1,2]

[1] N. N. Blokhin Russian Cancer Research Center, 24 Kashirskoe shosse, Moscow 115478, Russia;
[2] RUDN University, 6 Miklukho-Maklaya St., Moscow 117198, Russia
[3] I.M. Sechenov First Moscow State Medical University, 8-2 Trubetskaya St., Moscow 119991, Russia
[4] Mendeleev University of Chemical Technology of Russia, 9 Miusskaya ploshchad, Moscow 125047, Russia
[5] I.P. Pavlov Ryazan State Medical University, 9 Vysokovoltnaya st., Ryazan 390026, Russia

Table S1. Relative expression of the genes in HT29 cell line after treatment with LCS-1208 and LCS-1269 for 24 hours. Expression was upregulated or downregulated by 1.5 times or more in triplicate.

Signal pathway	Genes	Compounds		Signal pathway	Genes	Compounds	
		LCS-1208	LCS-1269			LCS-1208	LCS-1269
TGFß Pathway	ATF4			Angiogenesis	ANGPT1		
	CDKN1B		1.59		ANGPT2		
	EMP1				CCL2		
	GADD45B	1.93			FGF2		
	HERPUD1				FLT	1.82	
	IFRD1				KDR		0.47
	TNFSF10				PGF		
WNT Pathway	AXIN2	0.41	0.53		SERPINF1		
	CCND2				TEK		
	DAB2				VEGFC		0.41
	FOSL1			Apoptosis	APAF1		
	MMP7	4.20			BCL2L11	1.87	
	PPARD				BIRC3	2.38	2.79
	WISP1				CASP2		
NFκB Pathway	BCL2A1				CASP7	2.05	
	BIRC3				CASP9		
	CCL5	3.29	1.73		CFLAR		
	CSF1	2.85			FASLG		0.57
	ICAM1	2.61			NOL3		
	IFNG				XIAP		
	STAT1			Cell Cycle	AURKA		

	TNF	1.79			CCND2		
JAK/STAT Pathway	IRF1				CCND3		
	CEBPD				CDC20		
	LRG1				E2F4		
	MCL1	2.06			MCM2		
	SOCS3				MKI67		
	FCER2				SKP2		0.66
	GATA3				STMN1		
p53 Pathway	BAX				WEE1		
	BBC3			Cell Senescence	BMI1		
	BTG2				ETS2		
	CDKN1A				IGFBP3	0.16	0.54
	EGFR				IGFBP5		
	FAS				IGFBP7	0.41	
	GADD45A				MAP2K1		
	PCNA				MAP2K3		
	RB1				MAPK14		
Notch Pathway	HES1				SERPINB2		
	HES5				SOD1		
	HEY1				TBX2		
	HEY2			DNA Damage & Repair	DDB2		
	HEYL				DDIT3		
	ID1				ERCC3		
	JAG1	1.54			ERCC5		
	LFNG		0.43		GADD45G		0.34
	NOTCH1				LIG4		
Hedgehog Pathway	BCL2				POLB		
	BMP2				PPP1R15A		
	BMP4	1.59	1.87	EMT	CDH2		
	PTCH1				DSP		
	WNT1				FOXC2		
	WNT2B				GSC		
	WNT3A				KRT14		0.57
	WNT5A				OCLN		
	WNT6				SNAI1		
PPAR Pathway	ACSL3				SNAI2		
	ACSL4				SNAI3		
	ACSL5				SOX10		
	CPT2			Hypoxia	ADM		
	FABP1				ARNT		
	OLR1	3.34			CA9		
	SLC27A4				EPO		0.48

	SORBS1	0.31	0.39		HMOX1		
Oxidative Stress	FTH1				LDHA		
	GCLC				SLC2A1		
	GCLM			Metabolism	ACLY		
	GSR				ACSL4		
	NQO1				ATP5A1		
	SQSTM1				COX5A		
	TXN				CPT2		
	TXNRD1				G6PD		
Hypoxia	ADM				GPD2		
	ARNT				LPL		
	CA9	1.91			PFKL		
	EPO				UQCRFS1		
	LDHA	0.58	0.55	Telomeres & Telomerase	DKC1		
	SERPINE1				PINX1		
	SLC2A1				TEP1		
	VEGFA				TERF1		
TGFß Pathway and WNT Pathway	MYC				TERF2IP		
WNT Pathway and JAK/STAT Pathway	CCND1	0.37	0.47		TINF2		
NFκB Pathway and JAK/STAT Pathway	BCL2L1				TNKS		
Oxidative Stress and Hypoxia	HMOX1				TNKS2		

Table S2. The data on cytotoxicity of gold standard treatment compounds and LCSs in 11 tumor cell lines.

Cell line	Anticancer agent (Gold standard treatment)	Cytotoxicity (IC_{50})	Cytotoxicity of LCSs	
			LCS-1208	LCS-1269
MCF-7	Doxorubicin	0.267 µM [1]	5.5 µM	31 µM
	Cisplatin	9.7 µM [2]		
HepG2	Doxorubicin	0.478 µM [1]	1.7 µM	2.5 µM
U251	Temozolomide	176.5 µM [3]	0.36 µM	1.2 µM
A549	Cisplatin	6.59 µM [4]	1.0 µM	3.2 µM
	Doxorubicin	0.32 µM [5]		
PC-3	Docetaxel	0.0005 µM [6]	0.97 µM	24 µM
	Cisplatin	3.3 µM [7]		
HT29	Doxorubicin	750 nM [8]	0.13 µM	1.4 µM
	Cisplatin	75,7 µM [9]		
HeLa	Cisplatin	5.0 µM [2]	28,1	26,6
	Doxorubicin	0.374 µM [10]		
CCRF CEM	Cisplatin	0.7 µM [11]	2.0 µM	6.8 µM
Granta-519	Doxorubicin	212.7 nM (48 h) [12]	0.071 µM	0.60 µM
K562	Doxorubicin	About 1 µM [13]	6.0 µM	>500 µM
KG-1	Doxorubicin	About 9 µM (48 h) [14]	0.6 µM	7.1 µM

Figure S1. Analysis of the LCS-1269 effects on histone modifications, Western blot results.

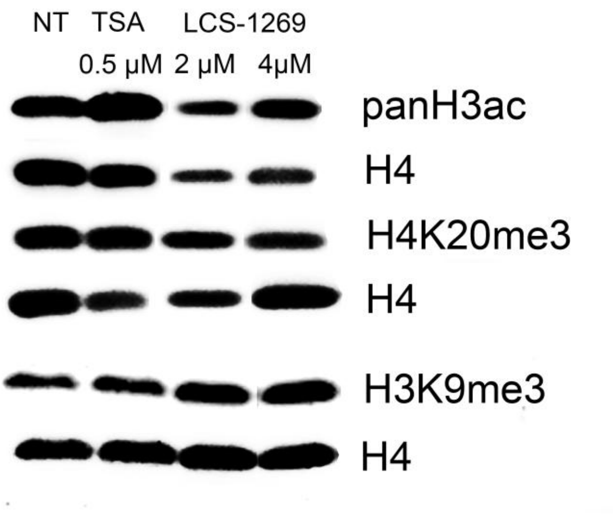

Figure S2. Analysis of the LCS-1269 effects on HDACs expression, Western blot results.

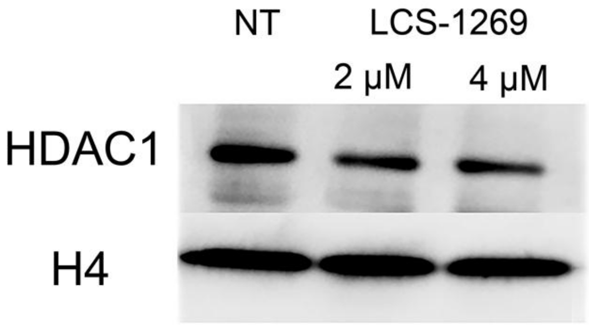

Figure S3. Analysis of the LCS-1269 effects on DNA methylation MSRE assay results.

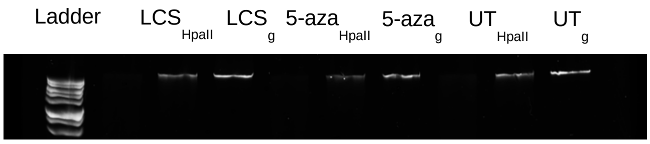

1. Kullenberg, F.; Degerstedt, O.; Calitz, C.; Pavlović, N.; Balgoma, D.; Gråsjö, J.; Sjögren, E.; Hedeland, M.; Heindryckx, F.; Lennernäs, H. In Vitro Cell Toxicity and Intracellular Uptake of Doxorubicin Exposed as a Solution or Liposomes: Implications for Treatment of Hepatocellular Carcinoma. *Cells* **2021**, *10*, doi:10.3390/cells10071717.
2. De Pascali, S.A.; Migoni, D.; Papadia, P.; Romano, A.; Marsigliante, S.; Pellissier, A.; Chardon-Noblat, S.; Ciccarese, A.; Fanizzi, F.P. New mononuclear and homodinuclear Pt(ii) complexes with heterocyclic nitrogen chelates: Synthesis, characterization, intercalating ability and in vitro cytotoxic activity evaluation. *Dalton Transactions* **2008**, 5911-5921, doi:10.1039/B807404F.
3. Poon, M.T.C.; Bruce, M.; Simpson, J.E.; Hannan, C.J.; Brennan, P.M. Temozolomide sensitivity of malignant glioma cell lines - a systematic review assessing consistencies between in vitro studies. *BMC cancer* **2021**, *21*, 1240, doi:10.1186/s12885-021-08972-5.

4. Davou, G.C.; Chuwang, N.; Essien, U.C.; Choji, T.P.; Echeonwu, B.; Lugos, M.D.J.I.R.J.o.M.; Sciences, M. Cytotoxicity analysis of etoposide and cisplatin on cell lines from human lung cancer and normal human lung. **2019**.
5. Kanintronkul, Y.; Worayuthakarn, R.; Thasana, N.; Winayanuwattikun, P.; Pattanapanyasat, K.; Surarit, R.; Ruchirawat, S.; Svasti, J. Overcoming multidrug resistance in human lung cancer with novel benzo[a]quinolizin-4-ones. *Anticancer research* **2011**, *31*, 921-927.
6. Makwana, V.; Dukie, A.S.; Rudrawar, S. Investigating the Impact of OGT Inhibition on Doxorubicin- and Docetaxel-Induced Cytotoxicity in PC-3 and WPMY-1 Cells. *International journal of toxicology* **2020**, *39*, 586-593, doi:10.1177/1091581820948433.
7. Altaf, M.; Casagrande, N.; Mariotto, E.; Baig, N.; Kawde, A.N.; Corona, G.; Larcher, R.; Borghese, C.; Pavan, C.; Seliman, A.A.; et al. Potent In Vitro and In Vivo Anticancer Activity of New Bipyridine and Bipyrimidine Gold (III) Dithiocarbamate Derivatives. *Cancers* **2019**, *11*, doi:10.3390/cancers11040474.
8. Atashpour, S.; Fouladdel, S.; Movahhed, T.K.; Barzegar, E.; Ghahremani, M.H.; Ostad, S.N.; Azizi, E. Quercetin induces cell cycle arrest and apoptosis in CD133(+) cancer stem cells of human colorectal HT29 cancer cell line and enhances anticancer effects of doxorubicin. *Iranian journal of basic medical sciences* **2015**, *18*, 635-643.
9. Leong, K.H.; Looi, C.Y.; Loong, X.M.; Cheah, F.K.; Supratman, U.; Litaudon, M.; Mustafa, M.R.; Awang, K. Cycloart-24-ene-26-ol-3-one, a New Cycloartane Isolated from Leaves of Aglaia exima Triggers Tumour Necrosis Factor-Receptor 1-Mediated Caspase-Dependent Apoptosis in Colon Cancer Cell Line. *PloS one* **2016**, *11*, e0152652, doi:10.1371/journal.pone.0152652.
10. Sadeghi-Aliabadi, H.; Minaiyan, M.; Dabestan, A. Cytotoxic evaluation of doxorubicin in combination with simvastatin against human cancer cells. *Research in pharmaceutical sciences* **2010**, *5*, 127-133.
11. Coronnello, M.; Marcon, G.; Carotti, S.; Caciagli, B.; Mini, E.; Mazzei, T.; Orioli, P.; Messori, L. Cytotoxicity, DNA damage, and cell cycle perturbations induced by two representative gold(III) complexes in human leukemic cells with different cisplatin sensitivity. *Oncology research* **2000**, *12*, 361-370, doi:10.3727/096504001108747828.
12. Tiemann, K.; Alluin, J.V.; Honegger, A.; Chomchan, P.; Gaur, S.; Yun, Y.; Forman, S.J.; Rossi, J.J.; Chen, R.W. Small interfering RNAs targeting cyclin D1 and cyclin D2 enhance the cytotoxicity of chemotherapeutic agents in mantle cell lymphoma cell lines. *Leukemia & lymphoma* **2011**, *52*, 2148-2154, doi:10.3109/10428194.2011.593272.
13. Li, J.; Liu, W.; Hao, H.; Wang, Q.; Xue, L. Rapamycin enhanced the antitumor effects of doxorubicin in myelogenous leukemia K562 cells by downregulating the mTOR/p70S6K pathway. *Oncology letters* **2019**, *18*, 2694-2703, doi:10.3892/ol.2019.10589.
14. Bahrami-Banan, F.; Sheikhha, M.H.; Ghasemi, N.; Majdizadeh, M.; Haghiralsadat, B.F. Preparation and Study of Nano-Niosomes Containing Doxorubicin and Evaluation of its Toxicity on Acute Myeloblastic Leukemia Cell Line KG-1 %J Payavard Salamat. **2018**, *12*, 309-323.
